# Daratumumab plus lenalidomide/bortezomib/dexamethasone in Black patients with transplant-eligible newly diagnosed multiple myeloma in GRIFFIN

**DOI:** 10.1038/s41408-022-00653-1

**Published:** 2022-04-13

**Authors:** Ajay K. Nooka, Jonathan L. Kaufman, Cesar Rodriguez, Andrzej Jakubowiak, Yvonne Efebera, Brandi Reeves, Tanya Wildes, Sarah A. Holstein, Larry D. Anderson, Ashraf Badros, Leyla Shune, Ajai Chari, Huiling Pei, Annelore Cortoos, Sharmila Patel, J. Blake Bartlett, Jessica Vermeulen, Thomas S. Lin, Paul G. Richardson, Peter Voorhees

**Affiliations:** 1 Winship Cancer Institute, Emory University, Atlanta, GA USA; 2 Wake Forest University School of Medicine, Winston-Salem, NC USA; 3 University of Chicago Medical Center, Chicago, IL USA; 4 OhioHealth, Columbus, OH USA; 5 University of North Carolina—Chapel Hill, Chapel Hill, NC USA; 6 Cancer & Aging Research Group, St. Louis, MO USA; 7 Division of Oncology & Hematology, Department of Internal Medicine, University of Nebraska Medical Center, Omaha, NE USA; 8 Simmons Comprehensive Cancer Center, UT Southwestern Medical Center, Dallas, TX USA; 9 Greenbaum Cancer Center, University of Maryland, Baltimore, MD USA; 10 Division of Hematologic Malignancies and Cellular Therapeutics, University of Kansas Medical Center, Kansas City, KS USA; 11 Tisch Cancer Institute, Mount Sinai School of Medicine, New York, NY USA; 12 Janssen Research & Development, LLC, Titusville, NJ USA; 13 Janssen Scientific Affairs, LLC, Horsham, PA USA; 14 Janssen Research & Development, LLC, Raritan, NJ USA; 15 Janssen Research & Development, LLC, Leiden, the Netherlands; 16 Dana-Farber Cancer Institute, Boston, MA USA; 17 Levine Cancer Institute, Atrium Health, Charlotte, NC USA

**Keywords:** Myeloma, Myeloma





Clinical outcomes and optimal therapy for Black patients with newly diagnosed multiple myeloma (NDMM) remain to be defined. In the United States, standard-of-care induction and consolidation regimens include lenalidomide, bortezomib, and dexamethasone (RVd) [1, 2]. The phase 2 GRIFFIN study (ClinicalTrials.gov Identifier: NCT02874742) evaluated the addition of the anti-CD38 monoclonal antibody daratumumab to RVd (D-RVd) induction and consolidation with lenalidomide (R) maintenance, in conjunction with autologous stem cell transplant (ASCT) in patients with NDMM in the United States [3]. The primary results of GRIFFIN were previously reported; D-RVd significantly improved the rate of stringent complete response (sCR) by the end of post-ASCT consolidation (D-RVd, 42.4% vs RVd, 32.0%; 1-sided *P* = 0.068 at the pre-specified 1-sided α level of 0.1), as well as the rates of minimal residual disease (MRD) negativity (10^−5^) [3]. Responses deepened with longer follow-up (median, 27.4 months); the rate of sCR continued to improve, and rates of MRD negativity (10^−5^) were also higher in the D-RVd group versus RVd group [4]. Here, we report a post hoc subgroup analysis of the GRIFFIN study examining the efficacy and safety of D-RVd in Black patients after all patients completed ≥12 months of maintenance therapy or discontinued at the median follow-up of 27.4 months.

The full study design of the randomized phase of the GRIFFIN study has previously been published [3]. Briefly, patients with NDMM who were eligible for ASCT received 4 cycles of D-RVd or RVd induction, high-dose therapy and ASCT, followed by 2 cycles of D-RVd or RVd consolidation, and D-R or R maintenance for up to 24 months. The primary endpoint was the sCR rate by the end of the post-ASCT consolidation treatment and was previously reported [3]. Secondary analyses were evaluated using 2-sided α of 0.05, not adjusted for multiplicity.

This analysis included 32 (D-RVd, *n* = 14 and RVd, *n* = 18) Black patients (15% of those enrolled) and 161 (D-RVd, *n* = 85 and RVd, *n* = 76) White patients (78% of those enrolled). Race was identified at study enrollment and recorded in the case report form; no Black patient self-identified with multiple races. Overall baseline demographics were previously published [3] and are shown by race in Table 1. Baseline characteristics were generally similar, except Black patients were slightly younger (median age: D-RVd, 58.5 years; RVd, 57.0 years) compared with White patients (D-RVd, 59.0 years; RVd, 61.5 years), and fewer Black males enrolled (D-RVd, 35.7% [*n* = 5]; RVd, 44.4% [*n* = 8]) compared with White males (D-RVd, 61.2% [*n* = 52]; RVd, 60.5% [*n* = 46]). Similar proportions of Black patients (D-RVd, 21.4% [*n* = 3]; RVd, 12.5% [*n* = 2]) and White patients (D-RVd, 15.0% [*n* = 12]; RVd, 13.7% [*n* = 10]) had high cytogenetic risk. Bone marrow involvement with ≥60% plasma cells was seen in a similar proportion of evaluable Black patients (D-RVd, 42.9% [*n* = 6]; RVd, 38.9% [*n* = 7]) and White patients (D-RVd, 43.5% [*n* = 37]; RVd, 36.8% [*n* = 28]).

**Table 1:** Baseline demographics and patient characteristics by race^a^.

Characteristic	Black		White	
	D-RVd (*n* = 14)	RVd (*n* = 18)	D-RVd (*n* = 85)	RVd (*n* = 76)
Age, years				
Median (range)	58.5 (29–67)	57.0 (48–67)	59.0 (35–70)	61.5 (41–70)
Category, *n* (%)				
<65 years	13 (92.9)	15 (83.3)	58 (68.2)	53 (69.7)
≥65 years	1 (7.1)	3 (16.7)	27 (31.8)	23 (30.3)
Sex, *n* (%)				
Male	5 (35.7)	8 (44.4)	52 (61.2)	46 (60.5)
Female	9 (64.3)	10 (55.6)	33 (38.8)	30 (39.5)
ECOG PS score, *n* (%)^b^	*n* = 13	*n* = 18	*n* = 84	*n* = 75
0	6 (46.2)	7 (38.9)	32 (38.1)	30 (40.0)
1	6 (46.2)	10 (55.6)	42 (50.0)	37 (49.3)
2	1 (7.7)	1 (5.6)	10 (11.9)	8 (10.7)
ISS disease stage, *n* (%)^c^				
I	9 (64.3)	11 (61.1)	40 (47.1)	37 (48.7)
II	3 (21.4)	4 (22.2)	32 (37.6)	27 (35.5)
III	2 (14.3)	3 (16.7)	12 (14.1)	10 (13.2)
Missing	0	0	1 (1.2)	2 (2.6)
Plasma cells, bone marrow biopsy/aspirate, *n* (%)^d^				
<10	3 (21.4)	0 (0.0)	6 (7.1)	6 (7.9)
10–59	5 (35.7)	11 (61.1)	40 (47.1)	38 (50.0)
≥60	6 (42.9)	7 (38.9)	37 (43.5)	28 (36.8)
Missing	0 (0.0)	0 (0.0)	2 (2.4)	4 (5.3)
Cytogenetic risk, *n* (%)^e^	*n* = 14	*n* = 16	*n* = 80	*n* = 73
Standard risk	11 (78.6)	14 (87.5)	68 (85.0)	63 (86.3)
High risk	3 (21.4)	2 (12.5)	12 (15.0)	10 (13.7)
Time since initial MM diagnosis (months)	*n* = 14	*n* = 18	*n* = 84	*n* = 75
Median (range)	0.6 (0–3)	0.7 (0–4)	0.7 (0–12)	0.9 (0–61)

D-RVd daratumumab plus lenalidomide/bortezomib/dexamethasone, RVd lenalidomide/bortezomib/dexamethasone, ECOG PS Eastern Cooperative Oncology Group performance status, ISS International Staging System, MM multiple myeloma.

^a^Demographics and clinical characteristics were based on electronic case report forms completed by study sites.

^b^ECOG PS is scored on a scale from 0 to 5, with 0 indicating no symptoms and higher scores indicating increasing disability.

^c^ISS disease stage is based on the combination of serum β_2_-microglobulin and albumin levels. Higher stages indicate more advanced disease.

^d^Highest value by biopsy or aspirate.

^e^Cytogenetic risk was assessed by fluorescence in situ hybridization (local testing); high risk was defined as the presence of del17p, t(4;14), or t(14;16) among patients with available cytogenetic risk data.

Treatment delivery was similar among randomized Black and White patients. The median lenalidomide relative dose intensities for Black patients were 81.6% (range, 48.1–100.0%) in the D-RVd group and 80.2% (range, 33.9–100.0%) in the RVd group. The median lenalidomide relative dose intensities among White patients were 87.7% (range, 26.1–101.6%) in the D-RVd group and 96.6% (range, 30.2–100.0%) in the RVd group. Lenalidomide cycle delays occurred in similar proportions of Black patients (D-RVd, 42.9% [*n* = 6]; RVd, 50.0% [*n* = 9]) and White patients (D-RVd, 43.4% [*n* = 36]; RVd, 45.9% [*n* = 34]). Similar proportions of Black patients (D-RVd, 14.3% [*n* = 2]; RVd, 50.0% [*n* = 9]) and White patients (D-RVd, 20.0% [*n* = 17]; RVd, 46.1% [*n* = 35]) discontinued study therapy; however, discontinuation rates were higher for both Black and White patients in the RVd group. Among Black patients, most patients discontinued therapy for the primary reason of withdrawal by patient (D-RVd, 0%; RVd, 16.7% [*n* = 3]) and adverse event (D-RVd, 7.1% [*n* = 1]; RVd, 11.1% [*n* = 2]). Among White patients, most patients discontinued therapy for the primary reason of progressive disease (D-RVd, 7.1% [*n* = 6]; RVd, 11.8% [*n* = 9]) and adverse event (D-RVd, 2.4% [*n* = 2]; RVd, 11.8% [*n* = 9]).

The rate of sCR by the end of post-ASCT consolidation was higher for the D-RVd group versus the RVd group in both Black patients (71.4% [*n* = 10] vs 33.3% [*n* = 6]; *P* = 0.0353) and White patients (42.7% [*n* = 35] vs 32.4% [*n* = 23]; *P* = 0.1923; Fig. 1A, B). With continued therapy, responses continued to deepened; after 12 months of maintenance therapy (median follow-up, 27.4 months), the rates of sCR were higher in the D-RVd versus RVd groups among both Black patients (85.7% [*n* = 12] vs 38.9% [*n* = 7], *P* = 0.0085) and White patients (62.2% [*n* = 51] vs 49.3% [*n* = 35], *P* = 0.1099). Notably, at last follow-up, the sCR rate doubled with the addition of daratumumab to RVd in Black patients, and 100% (*n* = 14) of Black patients who received D-RVd achieved complete response or better (≥CR) compared with 55.6% (*n* = 10) of Black patients who received RVd.

**Fig. 1:**
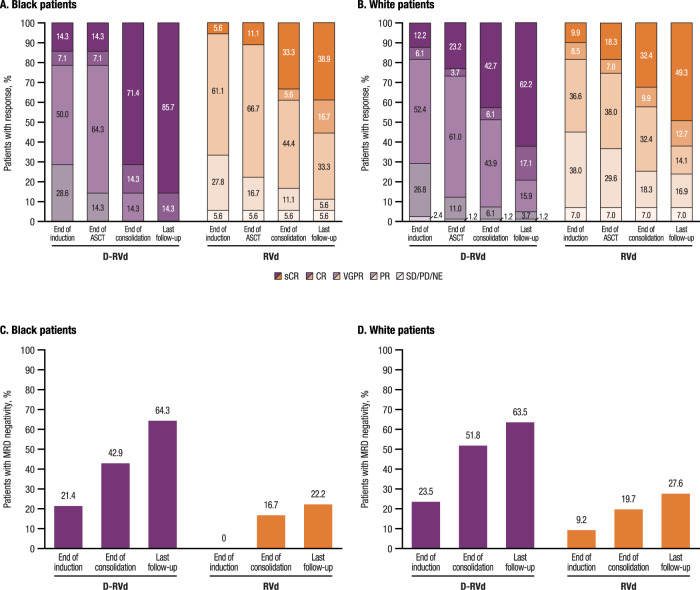
Summary of response rates and MRD-negativity (10^−5^) rates over time in Black and White patients. Response rates over time are shown for (A) Black patients (D-RVd, *n* = 14; RVd, *n* = 18) and (B) White patients (D-RVd, *n* = 82; RVd, *n* = 71) for the response-evaluable population, which included all randomized patients who had a confirmed diagnosis of multiple myeloma, measurable disease at baseline, received ≥1 dose of study treatment, and had ≥1 post-baseline disease assessment. Responses were assessed according to the IMWG criteria by computer algorithm, and rates of MRD negativity were measured by next-generation sequencing with a minimum sensitivity threshold of 1 in 10^5^ cells or higher, in accordance with IMWG criteria [13, 14]. MRD negativity testing occurred at baseline, first evidence of suspected CR or sCR, the end of induction and consolidation, and after 12 and 24 months of maintenance, regardless of response. Data analysis occurred at the median follow-up of 27.4 months, after all patients completed ≥12 months of maintenance therapy or discontinued. MRD-negativity (10^−5^) rates over time are shown for (C) Black patients (D-RVd, *n* = 14; RVd, *n* = 18) and (D) White patients (D-RVd, *n* = 85; RVd, *n* = 76) in the intent-to-treat population. Percentages may not equal 100 due to rounding. D-RVd daratumumab plus lenalidomide/bortezomib/dexamethasone; RVd lenalidomide/bortezomib/dexamethasone; ASCT autologous stem cell transplant; sCR stringent complete response; CR complete response; VGPR very good partial response; PR partial response; SD stable disease; PD progressive disease; NE not evaluable; IMWG International Myeloma Working Group; MRD minimal residual disease.

The MRD-negativity (10^−5^) rates at last follow-up were higher in the D-RVd group versus the RVd group among both Black patients (64.3% [*n* = 9] vs 22.2% [*n* = 4], *P* = 0.0293) and White patients (63.5% [*n* = 54] vs 27.6% [*n* = 21], *P* < 0.0001; Fig. 1C, D). The rate of MRD negativity (10^−6^) was also higher in the D-RVd group versus the RVd group for both Black patients (21.4% [*n* = 3] vs 5.6% [*n* = 1], *P* = 0.2951) and White patients (29.4% [*n* = 25] vs 11.8% [*n* = 9], *P* = 0.0070).

Median CD34^+^ cell yield among Black patients was 11.2 × 10^6^/kg for the D-RVd group and 9.4 × 10^6^/kg for the RVd group, and among White patients was 7.9 × 10^6^/kg for the D-RVd group and 9.4 × 10^6^/kg for the RVd group. The median number of CD34^+^ cells transplanted was similar for Black and White patients among treatment groups (Black: D-RVd, 4.9 × 10^6^/kg vs RVd, 4.8 × 10^6^/kg; White: D-RVd, 4.2 × 10^6^/kg vs RVd, 5.4 × 10^6^/kg), and hematopoietic reconstitution was comparable (median number of days for neutrophil engraftment [Black: D-RVd, 11.5 vs RVd, 11.5; White: D-RVd, 12.0 vs RVd, 11.5] and platelet engraftment [12.0 vs 13.0; 13.0 vs 12.0]).

The 3 most common treatment-emergent adverse events (TEAEs) of any grade for Black patients were upper respiratory tract infections (D-RVd, 78.6% [*n* = 11]; RVd, 50.0% [*n* = 9]), peripheral edema (64.3% [*n* = 9]; 50.0% [*n* = 9]), and peripheral neuropathy (57.1% [*n* = 8]; 66.7% [*n* = 12]) and the 3 most common for White patients were fatigue (72.3% [*n* = 60]; 60.8% [*n* = 45]), diarrhea (68.7% [*n* = 57]; 60.8% [*n* = 45]), and peripheral neuropathy (63.9% [*n* = 53]; 75.7% [*n* = 56]; Supplementary Table 1). Neutropenia was the most common TEAE of grade 3/4 in both Black patients (D-RVd, 50.0% [*n* = 7]; RVd, 22.2% [*n* = 4]) and White patients (43.4% [*n* = 36]; 18.9% [*n* = 14]), followed by lymphopenia in Black patients (28.6% [*n* = 4]; 38.9% [*n* = 7]) and also White patients (22.9% [*n* = 19]; 16.2% [*n* = 12]; Supplementary Table 2). Serious TEAEs were reported in Black patients with an incidence of 35.7% (*n* = 5) for D-RVd and 55.6% (*n* = 10) for RVd, with the most common being pneumonia (D-RVd, 21.4% [*n* = 3]; RVd, 16.7% [*n* = 3]). In White patients, serious TEAEs occurred in 43.4% (*n* = 36) of D-RVd patients and 48.6% (*n* = 36) of RVd patients; the most common was also pneumonia (D-RVd, 9.6% [*n* = 8]; RVd, 14.9% [*n* = 11]). TEAEs leading to treatment discontinuations in Black patients occurred in 35.7% (*n* = 5) of D-RVd patients and 27.8% (*n* = 5) of RVd patients. Among White patients, TEAEs leading to treatment discontinuation occurred in 19.3% (*n* = 16) and 23.0% (*n* = 17) of D-RVd and RVd patients, respectively. Peripheral neuropathy was the most common TEAE leading to discontinuation among both Black patients (D-RVd, 28.6% [*n* = 4]; RVd, 11.1% [*n* = 2]) and White patients (D-RVd, 3.6% [*n* = 3]; RVd, 5.4% [*n* = 4]), followed by neuralgia in Black patients (D-RVd, 7.1% [*n* = 1]; RVd, 5.6% [*n* = 1]) and upper respiratory tract infections (D-RVd, 2.4% [*n* = 2]; RVd, 1.4% [*n* = 1]) and pneumonia (D-RVd, 1.2% [*n* = 1]; RVd, 2.7% [*n* = 2]) in White patients. There were no other trends observed in TEAEs leading to treatment discontinuation (Supplementary Table 3). No deaths occurred due to TEAEs among Black patients, and 1 White patient in the D-RVd group had a TEAE leading to death. Infusion-related reactions occurred in 28.6% (*n* = 4) of Black D-RVd patients and 45.8% (*n* = 38) of White D-RVd patients, and the majority were grades 1/2.

Prior studies indicate disparities in outcomes for Black patients with multiple myeloma versus White patients [5, 6]; however, recent evidence suggests that Black and White patients can have comparable outcomes when Black patients are provided access to the same healthcare opportunities [5]. The present subgroup analysis of GRIFFIN indicates that Black patients can derive as great of a clinical benefit from the addition of daratumumab to RVd in the frontline setting as White patients and do not experience an increase in adverse events; these data have important implications for real-world practice [7], and in particular for specific toxicities, such as peripheral neuropathy [8]. Specifically, D-RVd versus RVd as induction and consolidation therapy improved depth of response, including rates of sCR and MRD negativity, in Black patients with NDMM. Additionally, continued treatment including daratumumab plus lenalidomide as maintenance therapy further improved depth of response. The efficacy outcomes and safety profiles of D-RVd in both Black and White patients were comparable and consistent with outcomes for the overall study population [3]. Although our analysis is limited by the sample size (32 Black patients total), these results suggest that Black patients with multiple myeloma experience similar outcomes as White patients when provided the same access to clinical studies and therapeutic options, underscoring the importance of appropriate representation of this patient population in clinical trials [9]. Historically, clinical study enrollment of Black patients has been low (~3%) in cancer clinical trials that led to cancer therapy approvals by the US Food and Drug Administration [10], particularly compared with Census data that estimate people of Black race to comprise 13% of the US population [11]. In GRIFFIN, Black patients comprised 15% of those enrolled, which marks a more accurate representation of this racial group in the general population as well as among multiple myeloma patients, 17% of whom are Black in the United States [12]. Despite this improvement, further studies enrolling larger numbers of Black patients are needed to confirm and better define the magnitude of daratumumab benefit in this patient population.

## Data sharing statement

The data sharing policy of Janssen Pharmaceutical Companies of Johnson & Johnson is available at https://www.janssen.com/clinical-trials/transparency. As noted on this site, requests for access to the study data can be submitted through Yale Open Data Access (YODA) Project site at http://yoda.yale.edu.

## Supplementary information

Supplementary Material
