# Supplementary material for: Daratumumab plus lenalidomide/bortezomib/dexamethasone in Black patients with transplant-eligible newly diagnosed multiple myeloma in GRIFFIN
Source: Blood Cancer J. 2022 Apr 13;12(4):63. doi: 10.1038/s41408-022-00653-1 (PMC9007985; doi:10.1038/s41408-022-00653-1)
Supplement: Supplementary file 2 — Supplementary Material [file 41408_2022_653_MOESM1_ESM.pdf]

Supplementary Material

This appendix has been provided by the authors to give readers additional information about their work.

Supplement to: AK Nooka, JL Kaufman, C Rodriguez, et al. Daratumumab plus lenalidomide/bortezomib/dexamethasone in Black patients with transplant-eligible newly diagnosed multiple myeloma: subgroup analysis of GRIFFIN

Supplementary Table 1. The most common any grade TEAEs (≥30%) in the safety analysis set.[a]

Patients with ≥1 TEAE, n (%)	Black		White	
	D-RVd (n=14)	RVd (n=18)	D-RVd (n=83)	RVd (n=74)
Hematologic				
Anemia	8 (57.1)	7 (38.9)	29 (34.9)	22 (29.7)
Neutropenia	8 (57.1)	6 (33.3)	51 (61.4)	25 (33.8)
Leukopenia	6 (42.9)	8 (44.4)	31 (37.3)	17 (23.0)
Thrombocytopenia	6 (42.9)	7 (38.9)	38 (45.8)	26 (35.1)
Lymphopenia	5 (35.7)	9 (50.0)	26 (31.3)	16 (21.6)
Nonhematologic				
Upper respiratory tract infection	11 (78.6)	9 (50.0)	55 (66.3)	38 (51.4)
Constipation	9 (64.3)	7 (38.9)	40 (48.2)	28 (37.8)
Peripheral edema	9 (64.3)	9 (50.0)	27 (32.5)	26 (35.1)
Peripheral neuropathy[b]	8 (57.1)	12 (66.7)	53 (63.9)	56 (75.7)
Fatigue	8 (57.1)	8 (44.4)	60 (72.3)	45 (60.8)
Nausea	8 (57.1)	9 (50.0)	41 (49.4)	37 (50.0)
Arthralgia	7 (50.0)	5 (27.8)	28 (33.7)	26 (35.1)
Cough	7 (50.0)	5 (27.8)	45 (54.2)	21 (28.4)
Headache	7 (50.0)	3 (16.7)	24 (28.9)	16 (21.6)
Insomnia	7 (50.0)	2 (11.1)	36 (43.4)	26 (35.1)
Vomiting	7 (50.0)	5 (27.8)	25 (30.1)	21 (28.4)
Diarrhea	6 (42.9)	6 (33.3)	57 (68.7)	45 (60.8)
Pyrexia	6 (42.9)	3 (16.7)	38 (45.8)	24 (32.4)
Rash maculo-papular	6 (42.9)	2 (11.1)	18 (21.7)	19 (25.7)
Back pain	5 (35.7)	9 (50.0)	33 (39.8)	21 (28.4)
Decreased appetite	5 (35.7)	2 (11.1)	18 (21.7)	8 (10.8)
Hypokalemia	5 (35.7)	6 (33.3)	21 (25.3)	16 (21.6)
Myalgia	5 (35.7)	4 (22.2)	21 (25.3)	14 (18.9)
Pain in extremity	5 (35.7)	7 (38.9)	15 (18.1)	14 (18.9)
Dysgeusia	4 (28.6)	6 (33.3)	19 (22.9)	12 (16.2)
Dyspnea	3 (21.4)	6 (33.3)	20 (24.1)	21 (28.4)
Dizziness	2 (14.3)	7 (38.9)	18 (21.7)	15 (20.3)
Hyperglycemia	1 (7.1)	6 (33.3)	12 (14.5)	11 (14.9)
Infusion related reactions	4 (28.6)	–	38 (45.8)	–

TEAE, treatment-emergent adverse event; D-RVd, daratumumab plus lenalidomide/bortezomib/dexamethasone; RVd, lenalidomide/bortezomib/dexamethasone.

[a]TEAEs occurring in ≥30% of the Black or White patients in the safety analysis set are shown.

[b]Peripheral neuropathy includes the preferred terms of peripheral neuropathy and peripheral sensory neuropathy.

Supplementary Table 2. The most common grade 3-4 TEAEs (≥20%) in the safety analysis set.[a]

Patients with ≥1 TEAE, n (%)	Black		White	
	D-RVd (n=14)	RVd (n=18)	D-RVd (n=83)	RVd (n=74)
Hematologic				
Neutropenia	7 (50.0)	4 (22.2)	36 (43.4)	14 (18.9)
Lymphopenia	4 (28.6)	7 (38.9)	19 (22.9)	12 (16.2)
Thrombocytopenia	4 (28.6)	2 (11.1)	11 (13.3)	6 (8.1)
Leukopenia	3 (21.4)	1 (5.6)	13 (15.7)	4 (5.4)
Nonhematologic				
Pneumonia	3 (21.4)	3 (16.7)	6 (7.2)	11 (14.9)

TEAE, treatment-emergent adverse event; D-RVd, daratumumab plus lenalidomide/bortezomib/dexamethasone; RVd, lenalidomide/bortezomib/dexamethasone.
[a]TEAEs of Grade 3 or 4 occurring in ≥20% of the Black or White patients in the safety analysis set are shown.

Supplementary Table 3. Summary of TEAEs leading to treatment discontinuation in the safety analysis set.

Patients with ≥1 TEAE, n (%)	Black		White	
	D-RVd (n=14)	RVd (n=18)	D-RVd (n=83)	RVd (n=74)
All TEAEs leading to discontinuation	5 (35.7)	5 (27.8)	16 (19.3)	17 (23)
Hematologic				
Neutropenia	0	1 (5.6)	1 (1.2)	1 (1.4)
Leukopenia	0	1 (5.6)	1 (1.2)	0
Lymphopenia	0	1 (5.6)	0	0
Anemia	0	1 (5.6)	0	0
Febrile neutropenia	0	0	1 (1.2)	0
Thrombocytopenia	0	0	0	1 (1.4)
Nonhematologic				
Peripheral neuropathy[a]	4 (28.6)	2 (11.1)	3 (3.6)	4 (5.4)
Neuralgia	1 (7.1)	1 (5.6)	0	1 (1.4)
Memory impairment	0	1 (5.6)	0	0
Cough	0	1 (5.6)	0	0
Dyspnea	0	1 (5.6)	0	0
Lacrimation increased	0	1 (5.6)	0	0
Constipation	0	1 (5.6)	0	0
Protein total decreased	0	1 (5.6)	0	0
Decreased appetite	0	1 (5.6)	0	0
Hyperglycemia	0	1 (5.6)	0	0
Hyperuricemia	0	1 (5.6)	0	0
Acute kidney injury	0	1 (5.6)	0	0
Dry skin	0	1 (5.6)	0	0
Upper respiratory tract infection	0	0	2 (2.4)	1 (1.4)
Cognitive disorder	0	0	1 (1.2)	0
Tremor	0	0	1 (1.2)	0
Pneumonia	0	0	1 (1.2)	2 (2.7)
Acute sinusitis	0	0	1 (1.2)	1 (1.4)
Cellulitis	0	0	1 (1.2)	0
Pneumonia bacterial	0	0	1 (1.2)	0
Sepsis	0	0	1 (1.2)	0
Diarrhea	0	0	1 (1.2)	1 (1.4)
Fatigue	0	0	1 (1.2)	0
Breast cancer	0	0	1 (1.2)	0

Cataract	0	0	1 (1.2)	0
Mood swings	0	0	1 (1.2)	0
Pruritus	0	0	1 (1.2)	0
Rash maculo-papular	0	0	1 (1.2)	0
Cerebrovascular accident	0	0	0	1 (1.4)
Seizure	0	0	0	1 (1.4)
Tinnitus	0	0	0	1 (1.4)
Peripheral edema	0	0	0	1 (1.4)
Hepatic cirrhosis	0	0	0	1 (1.4)
Serum sickness-like reaction	0	0	0	1 (1.4)
Nodular melanoma	0	0	0	1 (1.4)

TEAE, treatment-emergent adverse event; D-RVd, daratumumab plus lenalidomide/bortezomib/dexamethasone; RVd, lenalidomide/bortezomib/dexamethasone.

[a]Peripheral neuropathy includes the preferred terms of peripheral neuropathy and peripheral sensory neuropathy.
